# Soil Type Has a Stronger Role than Dipterocarp Host Species in Shaping the Ectomycorrhizal Fungal Community in a Bornean Lowland Tropical Rain Forest

**DOI:** 10.3389/fpls.2017.01828

**Published:** 2017-10-27

**Authors:** Adam L. Essene, Katherine L. Shek, J. D. Lewis, Kabir G. Peay, Krista L. McGuire

**Affiliations:** ^1^Department of Biological Sciences, Fordham University, New York City, NY, United States; ^2^Department of Biology, Institute of Ecology and Evolution, University of Oregon, Eugene, OR, United States; ^3^Department of Biology, Stanford University, Stanford, CA, United States

**Keywords:** tropical rain forest, mycorrhizal associations, ectomycorrhizal fungi, Dipterocarpaceae, host specificity

## Abstract

The role that mycorrhizal fungal associations play in the assembly of long-lived tree communities is poorly understood, especially in tropical forests, which have the highest tree diversity of any ecosystem. The lowland tropical rain forests of Southeast Asia are characterized by high levels of species richness within the family Dipterocarpaceae, the entirety of which has been shown to form obligate ectomycorrhizal (ECM) fungal associations. Differences in ECM assembly between co-occurring species of dipterocarp have been suggested, but never tested in adult trees, as a mechanism for maintaining the coexistence of closely related tree species in this family. Testing this hypothesis has proven difficult because the assembly of both dipterocarps and their ECM associates co-varies with the same edaphic variables. In this study, we used high-throughput DNA sequencing of soils and Sanger sequencing of root tips to evaluate how ECM fungi were structured within and across a clay–sand soil nutrient ecotone in a mixed-dipterocarp rain forest in Malaysian Borneo. We compared assembly patterns of ECM fungi in bulk soil to ECM root tips collected from three ecologically distinct species of dipterocarp. This design allowed us to test whether ECM fungi are more strongly structured by soil type or host specificity. As with previous studies of ECM fungi on this plot, we observed that clay vs. sand soil type strongly structured both the bulk soil and root tip ECM fungal communities. However, we also observed significantly different ECM communities associated with two of the three dipterocarp species evaluated on this plot. These results suggest that ECM fungal assembly on these species is shaped by a combination of biotic and abiotic factors, and that the soil edaphic niche occupied by different dipterocarp species may be mediated by distinct ECM fungal assemblages.

## Introduction

Mycorrhizal fungi provide the physiological link between soil nutrients and at least 80% of all terrestrial plant species (Wang and Qiu, 2006). A growing body of research demonstrates that mycorrhizal associations can influence plant community assembly and facilitate plant coexistence (van der Heijden et al., 1998; Bever, 2002; McKane et al., 2002; Reynolds et al., 2003). However, the majority of this research has taken place in temperate, herbaceous communities, which are easy to manipulate experimentally and grow over multiple generations. The role that mycorrhizal associations play in the assembly of long-lived tree communities is much less understood, especially in tropical forests, which have the highest tree diversity of any ecosystem (Leigh et al., 2004). In Neotropical rain forests, mycorrhizal fungal communities are mostly comprised of arbuscular mycorrhizal (AM) fungi (Janos, 1985), the earliest-evolved and widespread mycorrhizal associations across all terrestrial ecosystems (Smith and Read, 2010; Lewis, 2016). Ectomycorrhizal (ECM) fungal associations are less common, formed by ∼4.5% of tree species worldwide (Brundrett, 2009), and are generally associated with monodominant or co-dominant tree stands in Neotropical and African tropical rain forests (Torti et al., 2001; McGuire et al., 2008).

The mycorrhizal ecology in the lowland tropical rain forests of Southeast Asia is distinct from that of their Neotropical and African counterparts; the canopy in this region is characterized by high levels of species richness within the family Dipterocarpaceae (Ashton, 2009), and all 500+ species surveyed to date, including fossilized specimens, have been shown to form ECM associations (Lee et al., 2002; Ducousso et al., 2004; Brearley, 2012). These ECM associations have been proposed as one of the factors that originally facilitated and continued to maintain the familial dominance of Dipterocarpaceae (Torti et al., 2001; Brearley, 2012). ECM associations have also been suggested, but never tested, as a mechanism for maintaining the coexistence of so many closely related tree species in this family (Smits, 1994). This assumption is based on the fact that individual ECM trees can associate with dozens of different ECM fungal species (Lewis et al., 2008; Avolio et al., 2009), and each of these species can exhibit varying enzymatic capabilities and foraging strategies (Agerer, 2001), and segregate and partition resources between different soil horizons (Dickie et al., 2002; Baier et al., 2006; McGuire et al., 2013). The composite ECM fungal community on the roots of a single tree may be functionally distinct (McGuire et al., 2010), providing one tree access to a different pool of soil resources than a neighboring tree with different ECM fungal associates.

Many studies have investigated other biotic and abiotic factors besides ECM associations that influence the distribution and coexistence of dipterocarp species, in particular on the island of Borneo, which contains the highest dipterocarp richness recorded anywhere on earth: 276 species in 13 different genera (Maury-Lechon and Curtet, 1998). One striking ecological pattern in these lowland rain forests is the affinity many species of dipterocarp exhibit for specific soil types (Baillie et al., 1987; Paoli et al., 2006; Sukri et al., 2012). This specialization influences dipterocarp assembly at local (Davies et al., 2005), mesoscale (Paoli et al., 2006), and regional (Potts et al., 2002) scales, and dipterocarp species associated with different soil types have different physiological (Baltzer et al., 2005) and demographic (Russo et al., 2005) traits.

The extent and scale at which edaphic variables influence the biodiversity and distribution of trees has long been a contentious issue in Neotropical rain forests (Pitman et al., 1999; Hubbell, 2001; Condit et al., 2002; Phillips et al., 2003). The consistency with which this pattern has been observed in this region suggests that dipterocarps are strongly influenced by differences in nutrient availability (Bungard et al., 2002; Russo et al., 2005, 2008; Sukri et al., 2012). As ECM associations are the primary interface of dipterocarp nutrient acquisition, these associations could be important mediators of different dipterocarp species’ habitat preference observed across soil edaphic gradients.

Despite the significant role that ECM fungi may play in structuring dipterocarp composition, few studies have been conducted on mixed-dipterocarp ECM communities using DNA sequences to identify either the symbiont or host. The only intensive molecular survey of ECM diversity in a mixed-dipterocarp forest (Peay et al., 2010) found that changes in ECM community structure, similarly to above-ground dipterocarp community structure (Davies et al., 2005), correlated with differences in soil nutrient content. As distributions of ECM host taxa can also influence ECM fungal assembly (Bruns et al., 2002; Ishida et al., 2007; Morris et al., 2008), the influence of the soil abiotic environment can be confounded when ECM host taxa co-vary with the same edaphic variables as their ECM symbionts. In order to determine whether ECM fungi can mediate the habitat specialization of their dipterocarp hosts, the relative influence of the soil abiotic environment needs to be decoupled from host tree identity. A recent study conducted a reciprocal transplant of dipterocarp seedlings across soil types, and found no evidence for ECM-fungal host specificity (Peay et al., 2015). However, the seedling stage is a small portion of the host lifespan, and since ECM communities associated with a tree may change over time (Twieg et al., 2007), it is also important to look at these patterns with adult dipterocarps.

In this study, we used a high-throughput DNA sequencing approach to evaluate how ECM fungi are structured across a clay–sand soil nutrient ecotone in a mixed-dipterocarp rain forest in Malaysian Borneo. We first evaluated patterns of ECM fungal assembly in bulk soil on each side of the ecotone. Then, we collected ECM root tips from three ecologically distinct adult species of dipterocarp in the genus *Shorea*: *S. acuta* (Ashton), a sand specialist, *S. inappendiculata* (Burck), a clay specialist, and *S. almon* (Foxw.), a soil generalist found on both sides of the ecotone. This design allowed us to decouple the relative influence of host tree and soil type on ECM assembly, and enabled us to test whether ECM fungi were more strongly structured by soil or host specificity in adult trees.

## Materials and Methods

### Site Description

Lambir Hills National Park (hereafter, Lambir) is located about 30 km inland from the northern coast of Borneo, in the state of Sarawak, Malaysia (4°10′51″ N, 114°01′12.6″ E). Lambir is an aseasonal lowland tropical rain forest, with a daily temperature range of 24–36°C and an average annual rainfall of 3000 mm (Lee et al., 2002). This study was conducted on a 52-ha Forest Dynamics Plot established in 1992 by the Smithsonian Center for Tropical Forest Science (CTFS), the Sarawak Forestry Department and the Plant Ecology Laboratory of Osaka City University, Japan. The forest on the plot has never been commercially logged (Lee et al., 2002). The plot is divided into 1300 permanent 20 m^2^ quadrats (Yamakura et al., 1995). All living woody plants on the plot with DBH ≥ 1 cm are identified and mapped every 5 years. As of the 2012 census, there were more than 1000 identified tree species on the plot, which makes this one of the richest plots in terms of trees species in the CTFS network (Condit et al., 2002).

The Lambir Forest Dynamics Plot is roughly split 1:3 by an east–west escarpment that separates two distinct sedimentary formations, which give rise to very different soil chemistries and associated plant communities. Detailed descriptions of Lambir’s geomorphology are available elsewhere (Baillie et al., 1987; Lee et al., 2002). In brief, the parent substrate that forms the cuestas found on the more elevated northwestern side of the plot is sandstone, which yields sandy loams. This sandstone overlies an older layer of shale sediments, which are exposed at the southern and southeastern edges of the plot and give rise to more clayey soils (hereby referred to as “clay”). The sandy soils are relatively nutrient-poor, well-drained, and accumulate a thick humic layer and dense root mat at the soil surface, while the clay soils are nutrient rich, have a high water-retention capacity, and a very thin (>1 cm) organic surface layer with no root mat. Cluster analyses of tree species distributions relative to each soil type on the plot have shown that the majority of trees (86.6%) exhibited soil habitat specificity (Davies et al., 2005).

### Experimental Design

**FIGURE 1:**
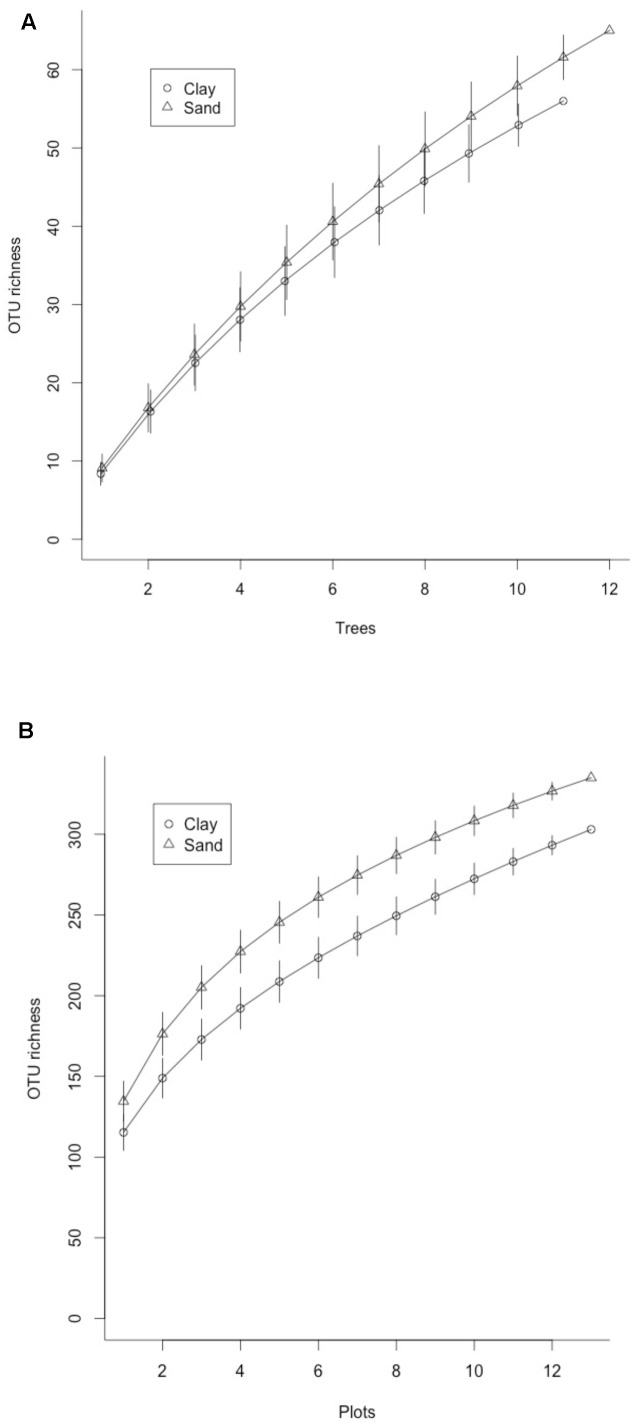
Rarefaction curves for ectomycorrhizal (ECM) fungal OTUs found in **(A)** bulk soil samples pooled from 12 plots on sand and clay soils in Lambir Hills National Park and **(B)** ECM fungal OTUs found on root tips sampled from three species of dipterocarp trees on sand and clay soils in Lambir Hills National Park. Error bars represent 95% confidence intervals for each iteration.

To estimate the local ECM fungal composition found on each soil type at Lambir, soil cores were collected from six 20 m × 20 m plots on the clay and sand sides of the ecotone in July 2010 (**Figure 1A**). In each plot, one 20 cm^2^ core was taken with an open-ended soil corer of 2.86 cm in diameter from each corner and the middle of the plot. All five soil cores were composited over a sterile 2 mm sieve to remove roots, stones, and organic detritus, and then stored at -20°C until analysis.

To evaluate the influence of different host species within and across the soil ecotone, we collected ECM root tips from three species of dipterocarp in the genus *Shorea*: a clay specialist, *S. inappendiculata*, a sand specialist, *S. acuta*, and a soil generalist, *S. almon* (Davies et al., 2005). We chose to focus on this genus because it contains the most species (55), has the highest basal area (487.8 m^2^), and largest number of the stems (23,813) in the plot (Lee et al., 2002). Individuals were randomly selected from Lambir’s 2012 tree census database with the following criteria: they had to be living, reproductively mature (Itoh et al., 2004), and more than 5 m from another tree. We used a partially crossed sampling design: five individuals of *S. inappendiculata* and six individuals of *S. acuta* were sampled from their respective soil habitat preference. Five and six individuals of *S. almon* were sampled on the sand and clay sides of the soil ecotone, respectively.

At each tree sampled for ECM root tips, four 10 cm^3^ cores of soil and roots were excavated with an EtOH-sterilized knife 0.5 m from the stem at each cardinal direction. The tree species of the roots within the cores were not confirmed, but sampling was conducted near the base of target trees to increase the likelihood of that species’ root capture. Root cores were then either gently rinsed with water to remove soil and examined on the day of collection, or soaked in water to prevent roots from drying out for no more than 24 h prior to examination. After most of the soil was rinsed off of each core, fine roots were removed and examined under a dissection microscope. ECM root clusters were identified visually, and a representative root tip was taken from each cluster in the order they were encountered. Although the target sample size was 20 root tips per tree, four of the trees had only 10, 19, 13, and 18, respectively (**Table 1**). ECM root tips were individually stored at room temperature in CTAB buffer until DNA extraction.

**Table 1:** Genera of ECM fungi observed in bulk soil samples using next-generation sequencing of the ITS1 regions using Illumina MiSeq.

Genus	Sand plots	Clay plots	Total
*Amanita*	5.97	1.09	3.57
*Amphinema*	0	0.11	0.05
*Apodus*	0	0.02	0.01
*Austropaxillus*	0.12	0.06	0.1
*Barssia*	0.06	0	0.03
*Boletus*	0.02	0.01	0.01
*Cantharellus*	0.1	0	0.05
*Clavulina*	0	0.04	0.02
*Cortinarius*	30.38	1.39	16.1
*Craterellus*	0.32	1.88	1.09
*Elaphomyces*	1.3	0.03	0.67
*Entoloma*	0.13	0	0.07
*Fimetariella*	0	0.08	0.04
*Gelasinospora*	0.25	0.03	0.14
*Helvella*	0.07	0	0.04
*Hydnellum*	0.08	0.02	0.05
*Hydnum*	0.02	0	0.01
*Hygrophorus*	0.11	0	0.06
*Inocybe*	4.99	5.33	5.16
*Lactarius*	1.62	25.96	13.61
*Lactifluus*	0	0.02	0.01
*Lasiosphaeria*	0.01	0.02	0.01
*Lyophyllum*	5.19	0	2.64
*Peziza*	0.35	0.4	0.37
*Phellodon*	0	0.02	0.01
*Piloderma*	1.18	0	0.6
*Podospora*	0.02	0.01	0.01
*Ramaria*	0	1.22	0.6
*Russula*	21.45	25.94	23.66
*Sarcodon*	0.85	0	0.43
*Scleroderma*	0	0.07	0.03
*Sebacina*	0.37	0.31	0.34
*Sphaerosporella*	23.34	27.83	25.55
*Thelephora*	0.37	0.02	0.2
*Tomentella*	1.27	7.93	4.55
*Tremellodendron*	0	0.06	0.03
*Tuber*	0	0.09	0.04


*The percent of sequences assigned to each genus is shown within each soil type*.

### Molecular Analyses

We identified the ECM fungal community in the soil core samples using a barcoded, high-throughput Illumina sequencing method previously described (McGuire et al., 2013). MoBio PowerSoil Extraction Kits (MO BIO Laboratories Inc., Carlsbad, CA, United States) were used to extract DNA from each soil sample. Extractions were done in triplicate to ensure a more complete characterization of microbial DNA from each sample (Feinstein et al., 2009). We targeted the internal transcribed spacer region 1 (ITS1) of ribosomal DNA using a modification of the fungal-specific primer pair ITS1F and ITS2 (White et al., 1990) adapted for the Illumina platform (McGuire et al., 2013). Both primers included Illumina adapter sequences, a 2-bp linker sequence and primer pad, with the 3′-primer (ITS2) also incorporating a 12-bp sample barcode sequence. PCR was conducted in 25 μl reactions containing 10 μM of each primer. PCR cycles were performed as follows: 94°C for 3 min, then 35 cycles of 94°C for 45 s, 50°C for 60 s, 72°C for 90 s, then 10 min at 72°C. PCR products (amplicon length ∼250 bp) were visualized using gel electrophoresis and successful reactions were quantified using the Quant-iT PicoGreen dsDNA assay (Thermo Fisher Scientific Inc., Waltham, MA, United States) with a spectrofluorometer. Sequencing was done at the New York Medical College (Valhalla, NY, United States) using an Illumina Miseq (Illumina Inc., San Diego, CA, United States). Sequences were deposited into GenBank under accession numbers MG018027-MG018198.

Raw sequences that Illumina generated from the bulk soil samples were demultiplexed using an in-house (University of Colorado) Python script and then processed following the UPARSE pipeline (Edgar, 2010). Demultiplexed reads were filtered by removing sequences with quality scores <23, dereplicating them, and then removing singletons and sequences <75% similar to any sequence in the UNITE database (Koljalg et al., 2005). A *de novo* database was constructed by clustering the remaining sequences into operational taxonomic units (OTUs) using a 3% sequence radius. Raw, demultiplexed sequences, rarified to 20,700 sequences per sample, were then mapped to this filtered database at a 97% similarity threshold to calculate sequence counts per OTU per soil sample. Taxonomy was assigned to each OTU using BLAST, and ECM fungi were identified from matches to known ECM taxa based on recent phylogenetic and stable isotope data (Tedersoo et al., 2010). Raw sequences were deposited in NCBI’s Sequence Read Archive (SRA) database under the accession number PRJNA413552.

To identify the ECM fungi associating with the root tip samples, we used a Sanger sequencing approach. We isolated DNA from each root tip using the Qiagen DNEasy Plant Mini Kit (Qiagen, Venlo, Netherlands) with a slightly modified protocol for tough plant tissue. Each root tip and its storage CTAB buffer were transferred to a 2 mL screw cap tube with 0.5 mm metal beads, and Qiagen-supplied lysis buffer was added to bring the total volume to 1 ml. Root tips were pulverized using a Mini Bead Beater 16 (Biospec Products Inc., Bartlesville, OK, United States) at max speed for 30 s increments until the root tissue was completely homogenized, and then lysate was incubated at 65°C for an hour. All subsequent steps adhered to the manufacturer’s guidelines. We amplified the ITS1 and ITS2 region of ribosomal RNA using the fungal-specific primer pair ITS1f and ITS4 (Gardes and Bruns, 1993). PCR was conducted in 20 μl reactions with 10 μM of each primer using the following cycle parameters: 95°C for 3 min, then 14 cycles at 95°C for 35 s, 55°C for 55 s, 72°C for 60 s, then 14 cycles at 95°C for 35 s, 55°C for 55 s, 72°C for 120 s, then 8 cycles at 95°C for 35 s, 55°C for 55 s, 72°C for 180 s, then 72°C for 10 min. PCR products (amplicon lengths ∼700 bp) were visualized using gel electrophoresis. Where multiple bands were visible, amplicons were isolated from the gel using either the GelElute Extraction Kit (5 Prime Inc., Gaithersburg, MD, United States) or the GeneJET Gel Extraction Kit (Thermo Fisher Scientific, Waltham, MA, United States). If multiple amplicons were within the target size range for the ITS region (500–700 bp), then each of the amplicons was gel purified. All successful reactions were sequenced in a single direction by the Beckman Coulter Genomics facility in Danvers, MA, United States, using the primer ITS1f.

Sequences from the root tip samples were manually edited with Geneious v. 7.3 (Biomatters, Auckland, New Zealand) to remove priming sites and low quality bases from the 5′ and 3′-ends of the reads. Sequences with quality scores <23 or reads shorter than 100 bp were excluded from further analysis. Remaining sequences were clustered into OTUs using a 3% sequence radius in USEARCH v. 8.0.1517 (Edgar, 2010). Chimeras were removed using both *de novo* and open-reference-based detection in USEARCH using the UNITE database as a reference (Koljalg et al., 2005). All root tip sequences were mapped to this filtered database with a 97% similarity cutoff to calculate sequence counts per OTU per tree. Taxonomy was assigned to each OTU using UBLAST with a database of ITS sequences curated from a previous study on the plot (Peay et al., 2010), sporocarps collected on the plot and identified by the Peay Lab (Stanford University, Stanford, CA, United States), and vouchered sporocarp specimens from the Forest Research Institute of Malaysia (FRIM) herbarium. To ensure accuracy, these taxonomic assignments were compared to the top BLAST hit on GenBank (Benson et al., 2009). The OTU sequences were then filtered to include only sequences that matched to known ECM lineages following Tedersoo et al. (2010).

### Statistical Analyses

Rarefaction curves for bulk soil and root tip ECM OTUs were constructed using the package BiodiversityR (Kindt and Kindt, 2015) in R version 3.20 (R Core Team, 2014). The Chao1 estimator was run for both root tips and bulk soil ECM fungi using the estimateR function of the Vegan package in R. To examine the relative influence of soil type and host species on ECM assembly, we used permutational analysis of variance (permANOVA, 10,000 permutations) (Anderson, 2001) and visualized results using non-metric multidimensional scaling (NMDS) ordination using Primer v. 6.1.13 with permANOVA+ v. 1.0.3 (Clarke and Gorley, 2006). Analysis of both bulk soil and root tip ECM fungal community structure was conducted using Bray–Curtis dissimilarity of square root-transformed OTU counts for each sample. For pairwise comparisons of the root tip ECM fungal communities found on each host species nested within the factor *Soil Type*, we excluded the one *S. inappendiculata* that was sampled on sandy soil because insufficient permutations (3) were possible to conduct a pairwise test with either *S. almon* or *S. acuta*.

## Results

Of the 2853 fungal OTUs recovered from the bulk soil cores, 197 were from established ECM lineages, assigned to 38 genera (**Table 1**). The most abundant genera were *Russula* (Russulaceae), *Cortinarius* (Cortinariaceae), *Lactarius* (Russulaceae), *Inocybe* (Inocybaceae), *Tomentella* (Thelephoraceae), and *Amanita* (Amanitaceae). The majority of the taxa recovered were present in only one soil type; 130 (65%) of the 197 taxa were only found on either sand or clay. Mean OTU richness was not significantly different in sand soils compared to clay (38.7 vs. 42.8). While rarefaction curves indicated that Illumina sequencing did not fully capture the total OTUs found in each soil type (**Figure 1**), the Chao1 estimator also did not predict significantly higher OTU richness in sand vs. clay soils (69.3 vs. 69.0, respectively).

**FIGURE 2:**
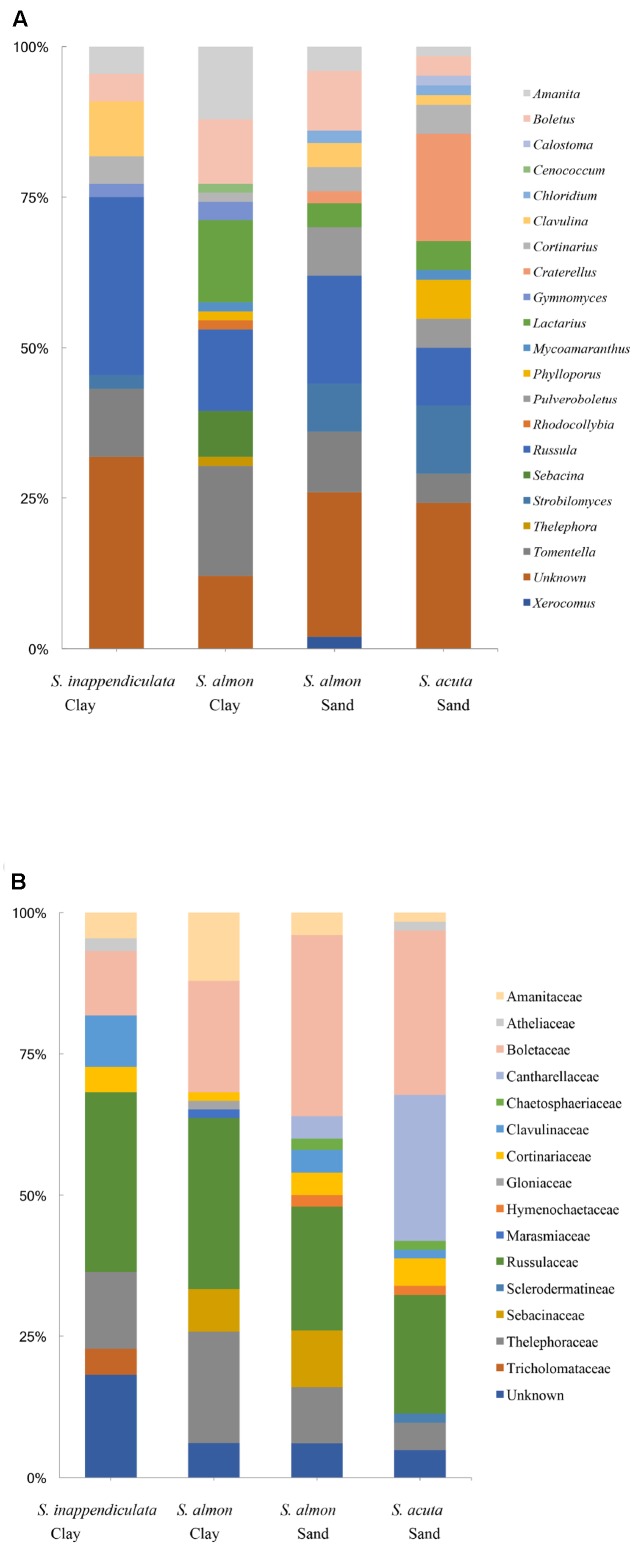
Genera **(A)** and families **(B)** of ECM fungi observed on root tip samples using Sanger sequencing of the ITS1 and ITS2 regions. Percent of sequences assigned to each genus is shown with respect to each host species and soil type.

Of the 193 OTUs recovered from the root tip samples, 112 were from established ECM lineages, assigned to 20 genera (**Figure 2A**) and 16 families (**Figure 2B**). The most common genera were *Russula* (Russulaceae), *Tomentella* (Thelephoraceae), *Boletus* (Boletaceae), *Lactarius* (Russulaceae) *Amanita* (Amanitaceae) and *Craterellus* (Cantharellaceae). The majority of the taxa detected in root tip samples had restricted distributions relative to soil type. Of the 112 taxa identified, 101 (90%) were found in root tips collected in only one soil type. The same was true for different host species; 98 (87.5%) of the taxa were found associating with only one species of *Shorea*, while 13 (11.5%) were found in multiple species, only two of which (1.7%) were found in all three. Ten of the 11 taxa found in only two species were shared between *S. almon* and *S. acuta*, and 9 of the 13 taxa found in multiple tree species were among the most common found in the plot. Observed OTU richness was not significantly higher for root tips collected from trees on sand soils than clay soils, although rarefaction curves did not show significant separation and indicate that we did not completely capture the diversity found in either side of the ecotone (**Figure 1B**). The Chao1 estimate also did not predict significantly higher ECM richness in sand soils (9.7) compared to clay soils (8.3).

**FIGURE 3:**
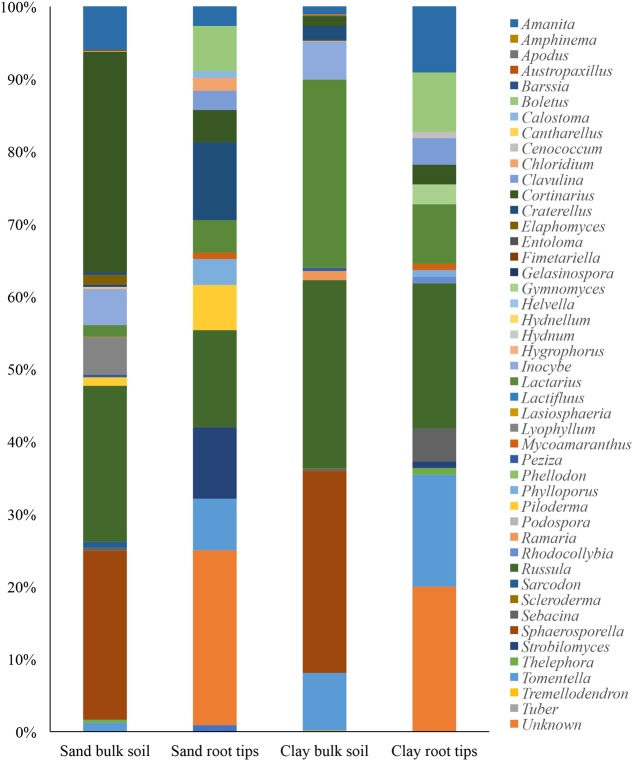
Genera of ECM fungal taxa detected in each soil type, with bulk soil taxa (from Illumina sequencing) and root tip taxa (from Sanger sequencing) aligned to see qualitative differences.

When we qualitatively compared the ECM taxa found in bulk soil versus on root tips, we found that 30% of the ECM genera were shared in the sand and 35% were shared in the clay (**Figure 3**). However, due to the difference in sequencing methodologies a formal statistical test could not be done.

**FIGURE 4:**
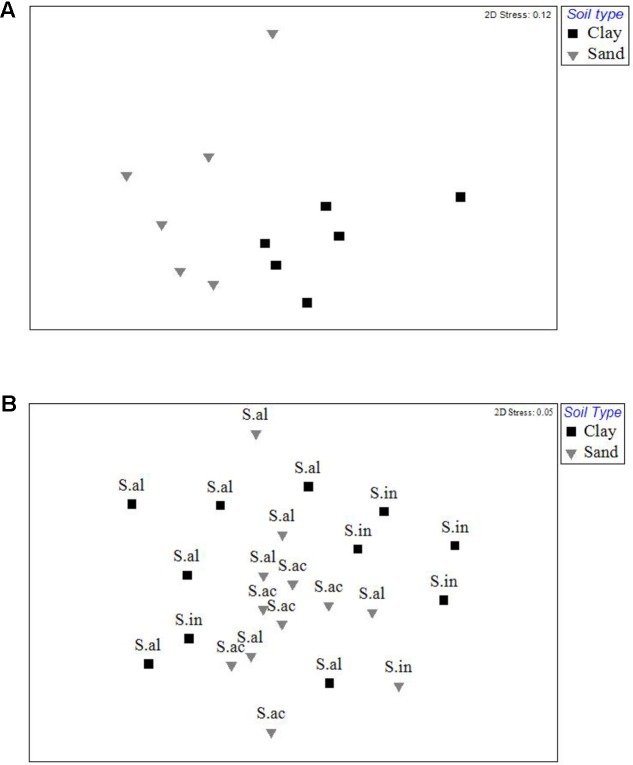
**(A)** Non-metric multidimensional scaling ordination of the ECM fungal communities found on two different soil types at Lambir Hills National Park. Each point represents pooled OTU counts from six different 400 m^2^ plots on each soil type. Distance between points represents rank dissimilarity using a Bray–Curtis index. **(B)** Non-metric multidimensional scaling ordination of ECM fungal communities found on root tips collected from three species of dipterocarps at Lambir Hills National Park. Each point represents the pooled OTU counts from an individual tree. Distance between points represents rank dissimilarity using a Bray–Curtis index. Letters denote samples from individual tree species (S.al = *Shorea almon*, S.in = *Shorea inappendiculata*, S.ac = *Shorea acuta*).

Analysis by permANOVA indicated that ECM fungal communities in both the bulk soil and root tip samples were significantly structured by soil type (**Figures 4A,B**). The global test of the effect of host species nested within soil type on the ECM root tip community was marginally insignificant (*p* = 0.054), yet pairwise comparisons revealed significant clustering was due to differences between *S. almon* and *S. inappendiculata* on the clay side of the ecotone (*p* = 0.03), but not between *S. almon* and *S. acuta* on the sand side of the ectone (*p* = 0.67). This host effect is illustrated by the two different clusters of root tip samples from the clay side of the plot in the NMDS ordination (**Figure 4B**).

## Discussion

We found strong edaphic specialization across the soil ecotone for both the root tip and bulk soil components of ECM fungal communities, supporting the hypothesis that soil type is the primary determinants of ECM fungal community assembly. The strong edaphic segregation of ECM fungal communities in this study is consistent with previous work at Lambir Hills finding that ECM fungi exhibit sharp compositional shifts across clay and sand soil types (Peay et al., 2010, 2015). Nonetheless, differences in ECM community composition were detected between two dipterocarp species within the same soil type, suggesting that host tree identity also plays a role in structuring ECM fungal communities, albeit a much weaker role than soil edaphic properties.

Our detection of ECM fungal host specificity in this study is in contrast with previous results from this dipterocarp forest (Peay et al., 2015). Specifically, a recently published reciprocal seedling transplant study conducted at Lambir Hills found no effect of host species identity on ECM fungal composition in 13 different dipterocarp seedling roots, but found significant structuring of ECM fungal composition across clay and sand soil types (Peay et al., 2015). The conflicting results from the current study may be due to the fact that adult ECM fungal communities are not reflective of seedling ECM fungal communities, as ECM fungal assembly in an individual tree may change as the host ages (Nara et al., 2003). In temperate forests, ECM fungi fall along a continuum of host specialization (Horton and Bruns, 1998), and there is some evidence that ECM fungal composition diverges with greater phylogenetic distance between host species (Ishida et al., 2007). While we only sampled three *Shorea* species, ECM fungal composition was only distinct in the two *Shorea* species that were less phylogenetically related (Ashton, 2003). More experimentation will be necessary to determine if the differences we observed in the ECM assembly of *S. almon* and *S. inappendiculata* is an example of strict host specificity, i.e., related to host–fungus genetic compatibility (Bruns et al., 2002), the result of indirect host effects via the nutrient content of leaf litter input (Cullings et al., 2003; Uriarte et al., 2015), or other host-related modifications to the local abiotic environment (Dickie et al., 2002).

Some ECM lineages exhibited consistent segregation across soil types at the level of both root tip and bulk soil, suggesting there are underlying physiological differences in these taxa that may drive their community assembly across the ecotone. In temperate and boreal ecosystems, differences in ECM assembly are often correlated with soil characteristics such as nitrogen content, water retention, pH, and cation exchange capacity (Lilleskov et al., 2002; Toljander et al., 2006; Piculell et al., 2008; Avolio et al., 2009; Polme et al., 2013) and these patterns may reflect a variety of physiological optima for different fungal taxa (Smith and Read, 2010). Different lineages of ECM fungi have also been shown to exhibit varying enzymatic capabilities and foraging strategies, or so-called “exploration types” (Agerer, 2001, 2006), and taxa with similar exploration types have been observed to respond similarly to environmental gradients of nitrogen (Lilleskov et al., 2002) and carbon (Markkola et al., 2004). Given the general taxonomic similarity between the ECM fungal communities found at Lambir and those observed in temperate and boreal forests (Peay et al., 2010), the mechanisms responsible for driving these differences are likely similar to those found elsewhere.

The greater relative abundance of Thelephoraceae in clay soils and *Cortinarius* in sandy soils is consistent with a previous study at this site (Peay et al., 2015), implying that these patterns are robust and not simply due to sampling bias or seasonal variation. While the specific mechanisms driving the assembly of taxa within each soil type require further study, one potential explanation is related to the different hyphal exploration types associated with each of these lineages. There is evidence that some species of *Cortinarius* form medium-range rhizomorphs specialized for the acquisition of organic N from leaf litter and humus, and are sensitive to increased availability of mineral N (Lilleskov et al., 2011). These taxa would be expected to provide a competitive advantage to hosts growing in oligotrophic soils rich in organic matter, such as the sandy soils found at Lambir Hills. Likewise, some taxa in the family Thelephoraceae form short, hydrophilic hyphae that may be favored by hosts in soils with greater labile nutrient availability, such as clay.

While edaphic filtering appeared to be the strongest community assembly mechanism for ECM fungi both in bulk soil and in root tips, the compositions of these two fractions were distinct, most notably with respect to the paucity of Boletaceae detected in the bulk soil samples. This finding contrasts with both the root tip samples and previous ECM surveys conducted in the plot, where taxa in this family were among the most abundant. This discrepancy may be due to the tendency of this lineage to form long-distance exploratory rhizomorphs (Agerer, 2001), which may have been excluded during the removal of roots that were sieved from the bulk soil sampling. Similarly, there is general evidence that taxa can differ in their investment into different structures such as root mantles, fruiting bodies, or extraradical hyphae (Gardes and Bruns, 1996; Koide et al., 2005). Another notable difference between the root tip and bulk samples is the greater relative abundance of ECM Ascomycota in the bulk soil samples, which may reflect a greater proportion of Ascomycota hyphal or propagule biomass in the soil rather than in mycorrhizal association with roots. Differential biomass allocation between fruiting bodies, ectomycorrhizae, extraradical hyphae, and propagules has been observed previously in both primary forests (Gardes and Bruns, 1996; Taylor and Bruns, 1999) and pine plantations (Koide et al., 2005). It is also possible that these results are simply due to differences in sequencing depth, as the root tip samples were analyzed by Sanger sequencing and the bulk soil samples were sequenced on the Illumina platform.

The results of this study suggest that ECM fungal assembly at Lambir is shaped by a combination of biotic and abiotic factors. Like previous ECM fungal studies conducted in the plot, both the bulk soil and root tip fractions of ECM communities were strongly structured by the soil differences between the clay and sand sides of the soil ecotone. Given the fact that differences in ECM community composition have been previously observed at Lambir (Peay et al., 2010), it appears that this result is robust and likely reflects some functional or physiological differences between the ECM taxa found in either side of the soil ecotone. Although the mechanisms remain unclear, the differences that we observed between the ECM fungal assemblies found in two of the three species of *Shorea* suggest that the soil edaphic niche occupied by some dipterocarp species may be mediated by distinct ECM fungal assemblages. Additional studies that incorporate a broader phylogenetic range of dipterocarp host species and include measurements of host-associated soil physiochemical modifications would enable a more mechanistic evaluation of this hypothesis.

## Author Contributions

Sampling design was conceived by AE, JL, KP, and KM. Laboratory and data analyses were done by AE, KS, and KM, and manuscript writing was completed by all authors.

## Conflict of Interest Statement

The authors declare that the research was conducted in the absence of any commercial or financial relationships that could be construed as a potential conflict of interest.
